# Beyond dysbiosis: microbial metabolites as key remodelers of nasal mucosal immune tolerance in chronic rhinosinusitis

**DOI:** 10.3389/fimmu.2026.1831258

**Published:** 2026-06-16

**Authors:** Guan-Jiang Huang, Zi-Qing Chen, Chao-Qing Long, Qi-Ping Luo, Zhi-Jun Fan, Biao-Qing Lu

**Affiliations:** 1 Department of Otorhinolaryngology Head and Neck Surgery, Zhongshan Hospital of Traditional Chinese Medicine, Affiliated to Guangzhou University of Chinese Medicine, Zhongshan, Guangdong, China; 2 The Tenth Clinical Medical College of Guangzhou University of Chinese Medicine, Zhongshan, Guangdong, China

**Keywords:** chronic rhinosinusitis, immune tolerance, microbial metabolites, nasal mucosa, postbiotics

## Abstract

Chronic rhinosinusitis (CRS) is a heterogeneous inflammatory disorder of the nasal and paranasal sinus mucosa affecting approximately 11% of adults worldwide. Although compositional dysbiosis of the sinonasal microbiome has historically dominated etiological discourse, this framework inadequately accounts for the mechanistic complexity of mucosal immune dysregulation in CRS. Emerging evidence positions microbial metabolites, rather than microbial identity per se, as the proximate immunological mediators of commensal microbiota–host crosstalk. This review presents a comprehensive analysis of the “microbial metabolite-immune receptor axis” in CRS, encompassing three classes of protective commensal metabolites and their mechanisms of action. Short-chain fatty acids (SCFAs) activate GPR43 and inhibit histone deacetylases (HDACs) to induce FoxP3+ regulatory T cells (Tregs) and promote ILC3-derived IL-22 production. Tryptophan-derived indole metabolites engage the aryl hydrocarbon receptor (AhR) to sustain ILC3 barrier-protective function and suppress Th2/Th17 polarization. Secondary bile acids signal through FXR and TGR5 to modulate the Treg/Th17 balance. In contrast, virulence factors produced by Staphylococcus aureus (the dominant pathobiont in CRSwNP) drive NLRP3 inflammasome activation, macrophage pyroptosis, and epithelial tight junction disruption. The gut–nose metabolite axis further establishes that systemic depletion of gut-derived protective metabolites amplifies nasal mucosal immune dysfunction. Building upon this mechanistic framework, we propose postbiotic supplementation, defined as the direct administration of purified bioactive metabolites, as a precision therapeutic strategy to restore nasal mucosal immune homeostasis. Endotype-specific metabolite candidate selection, guided by individual patient metabolomics profiling, is central to this therapeutic approach.

## 1 Introduction

Chronic rhinosinusitis (CRS) is defined as persistent symptomatic inflammation of the nasal cavity and paranasal sinuses lasting at least 12 weeks, encompassing the phenotypic subgroups of CRS with nasal polyps (CRSwNP) and CRS without nasal polyps (CRSsNP) (1–3). With a global prevalence estimated at 10.9% among adults in Europe and comparable figures across North America and Asia, CRS represents a major public health burden associated with considerable impairment of olfaction, sleep quality, cognitive function, and overall quality of life (3–5). Despite advances in pharmacological management, including topical and systemic corticosteroids, functional endoscopic sinus surgery, and targeted biological therapies such as anti-IL-4Rα (dupilumab), anti-IL-5 (mepolizumab), and anti-IgE (omalizumab), post-treatment disease recurrence rates remain unacceptably high (2, 6, 7). These persistent recurrence rates underscore the critical need to interrogate the fundamental pathogenic mechanisms sustaining mucosal inflammation.

The immunological landscape of CRS is highly complex. CRSwNP is predominantly driven by type 2 inflammation characterized by IL-4, IL-5, and IL-13 overproduction, IgE class switching, eosinophilic tissue infiltration, and goblet cell hyperplasia, whereas CRSsNP more commonly exhibits type 1 or type 3 neutrophilic inflammation (3, 8, 9). At the cellular level, impaired Treg activity, dysregulated innate lymphoid cell (ILC) responses, and progressive epithelial barrier dysfunction collectively sustain the chronic inflammatory state (10). A defining immunological abnormality in CRS (particularly in CRSwNP) is the loss of nasal mucosal immune tolerance, wherein the sinonasal epithelium transitions from a homeostatic immunological interface into a chronically primed platform for exaggerated type 2 responses (11).

For two decades, the sinonasal microbiome was investigated primarily through a compositional lens. Culture-independent 16S rRNA amplicon sequencing revealed that CRS is characterized by depletion of commensal diversity and enrichment of potential pathogens (a state collectively designated as dysbiosis) (12–14). However, this compositional framework increasingly faces conceptual limitations, as identical taxon-level changes correlate poorly with disease severity, and clinical outcomes are only weakly predicted by microbial community structure alone (15, 16). Probiotic interventions designed to restore microbiome composition have shown inconsistent clinical benefits, suggesting that the mere presence or absence of specific taxa is insufficient to explain the immunological aberrations of CRS.

Basic microbiome science has produced a transformative conceptual advance, demonstrating that commensal bacteria influence the host immune system not through their presence alone, but through the bioactive small molecules they secrete (17–19). Microbial metabolites, including SCFAs, tryptophan-derived indoles, secondary bile acids, and polyamines, engage specific host cell receptors including G protein-coupled receptors (GPCRs), nuclear receptors, and ligand-activated transcription factors to regulate immune cell differentiation, cytokine production, and epithelial barrier function (18–20). This “metabolite-immune receptor axis” framework reconceptualizes the primary investigational objective from the identification of microbial taxa to the characterization of bioactive metabolites produced and the receptor pathways they activate, an approach that is both mechanistically tractable and therapeutically actionable.

**Figure 1:**
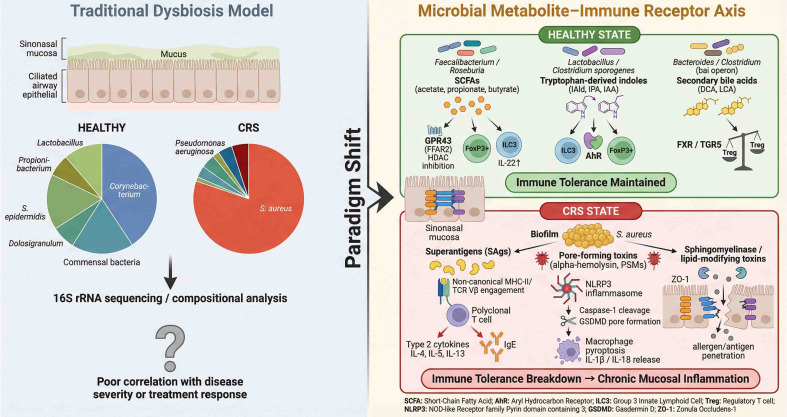
Paradigm shift from the traditional compositional dysbiosis model to the microbial metabolite–immune receptor axis in CRS. Left panel: 16S rRNA sequencing reveals depletion of commensals (*Lactobacillus*, *Corynebacterium*, *Dolosigranulum*) and enrichment of *S. aureus* in CRS. However, taxon-level changes correlate poorly with disease severity or treatment response. Right panel: In healthy individuals, SCFAs, tryptophan-derived indoles, and secondary bile acids collectively maintain immune tolerance. In CRS, *S. aureus*-derived superantigens, pore-forming toxins (NLRP3/pyroptosis), and sphingomyelinase disrupt tight junctions, driving immune tolerance breakdown and chronic mucosal inflammation. SCFA, short-chain fatty acid. NLRP3, NOD-like receptor family Pyrin domain containing 3.

This review synthesizes current mechanistic evidence supporting the microbial metabolite-immune axis in CRS, drawing upon foundational discoveries from gut mucosal immunology and translating these principles to the nasal mucosal context. We describe three classes of commensal-derived protective metabolites and their receptor signaling mechanisms. We further examine how pathobiont-derived metabolites and toxins actively subvert nasal mucosal tolerance, explore the systemic dimension of the gut-nose metabolite axis, and propose postbiotic intervention as a precision therapeutic strategy to restore immune homeostasis. This framework reframes CRS management from broad-spectrum immunosuppression toward mechanism-driven restoration of metabolite–receptor signaling in the nasal mucosa (**Figure 1**).

## 2 The nasal sinus microbiome: from compositional dysbiosis to functional metabolomics

### 2.1 Microbial ecology of the healthy sinonasal niche

The healthy sinonasal microbiome is substantially less diverse than the gut microbiome but functionally active in producing metabolites that interact with sinonasal epithelial cells and resident immune populations. Culture-independent surveys of middle meatus and sinus mucosal specimens from healthy adults consistently identify a core microbiome dominated by *Corynebacterium*, *Dolosigranulum*, *Staphylococcus epidermidis*, *Propionibacterium*, and *Lactobacillus* species (12, 21–23). These commensals maintain ecological stability through the production of bacteriocins, H_2_O_2_, and lactic acid that competitively exclude pathogenic organisms, as well as through metabolite-mediated modulation of innate immune tone (24, 25). *Dolosigranulum pigrum* and *Corynebacterium accolens* are particularly notable for their capacity to suppress *S. aureus* colonization and to promote interferon signaling in nasal epithelial cells (26, 27). The composition of this core microbiome is dynamically regulated by host genetics, diet, environmental exposures, and prior antibiotic use, all of which influence the metabolite-producing capacity of the community (28).

The healthy sinonasal microbiome also encompasses a distinct fungal community (the mycobiome), principally comprising Malassezia, Aspergillus, Candida, and Cladosporium species co-residing alongside bacterial commensals (29, 30). Although immunological mechanisms remain incompletely understood, CRS patients may exhibit differential immune responses to airborne fungi relative to healthy individuals. The functional metabolic interactions between the sinonasal mycobiome and bacteriome represent an undercharacterized but increasingly recognized dimension of sinonasal microbial ecology directly relevant to the metabolite-immune receptor axis (31, 32).

### 2.2 Compositional shifts in CRS and the limits of taxon-centric analysis

Comparative 16S rRNA sequencing studies have consistently demonstrated that the CRS sinonasal microbiome is characterized by depletion of commensal organisms and enrichment of potential pathogens including *S. aureus*, *Pseudomonas aeruginosa*, and Enterobacteriaceae (17, 19, 33). *S. aureus* is particularly prevalent in CRSwNP, where it establishes biofilm communities that resist immune clearance and antibiotic treatment (33, 34). These compositional observations are scientifically important but clinically insufficient: neither the specific taxa altered in CRS nor their quantitative abundances robustly predict disease endotype, severity, or treatment response. The functional output of the microbiome, specifically its metabolite production capacity, ultimately connects microbial community structure to mucosal immune outcomes (15).

Cope et al. provided a landmark demonstration of this principle, showing that CRS patients harbor compositionally and functionally distinct sinus microbiota, and that these functional differences, rather than compositional ones, are the primary determinants of divergent immunological and clinical consequences (35). This finding supports a transition from taxon cataloguing toward metabolic function as the primary analytical target in CRS microbiome research.

### 2.3 Multi-omics integration: metagenomics and metabolomics in CRS

The limitations of compositional analysis have catalyzed adoption of complementary functional approaches. Metagenomic shotgun sequencing captures microbial functional gene content, including biosynthetic pathways for SCFA synthesis (butyrate kinase, propionate-CoA transferase), tryptophan catabolism (tryptophan lyase, IDO), and bile acid modification (7α-dehydroxylase) (20, 36). This approach thereby bridges the gap between taxonomic composition and metabolic function. Integration with untargeted metabolomics using liquid chromatography–mass spectrometry (LC-MS) or nuclear magnetic resonance (NMR) enables direct detection of the actual metabolite landscape in nasal secretions, sinus tissue, and systemic circulation, allowing correlation with immune phenotypes and clinical parameters (35, 37).

As Chang PV reviewed comprehensively, the mechanistic connection between gut microbiota and host physiology runs primarily through metabolite–receptor interactions that engage defined downstream signaling pathways (19). Malczewski et al. further demonstrated that unique metabolic signatures are associated with both favorable and unfavorable immunological outcomes in multiple inflammatory disease contexts, establishing the diagnostic and therapeutic potential of metabolomics-driven microbiome analysis (37). Cumulative evidence now supports a fundamental paradigm transition in the field, from cataloguing microbial taxa to deciphering the metabolite-mediated mechanisms through which microbiota communicate with the nasal mucosal immune system.

## 3 Protective microbial metabolites and nasal mucosal immune tolerance

**Figure 2:**
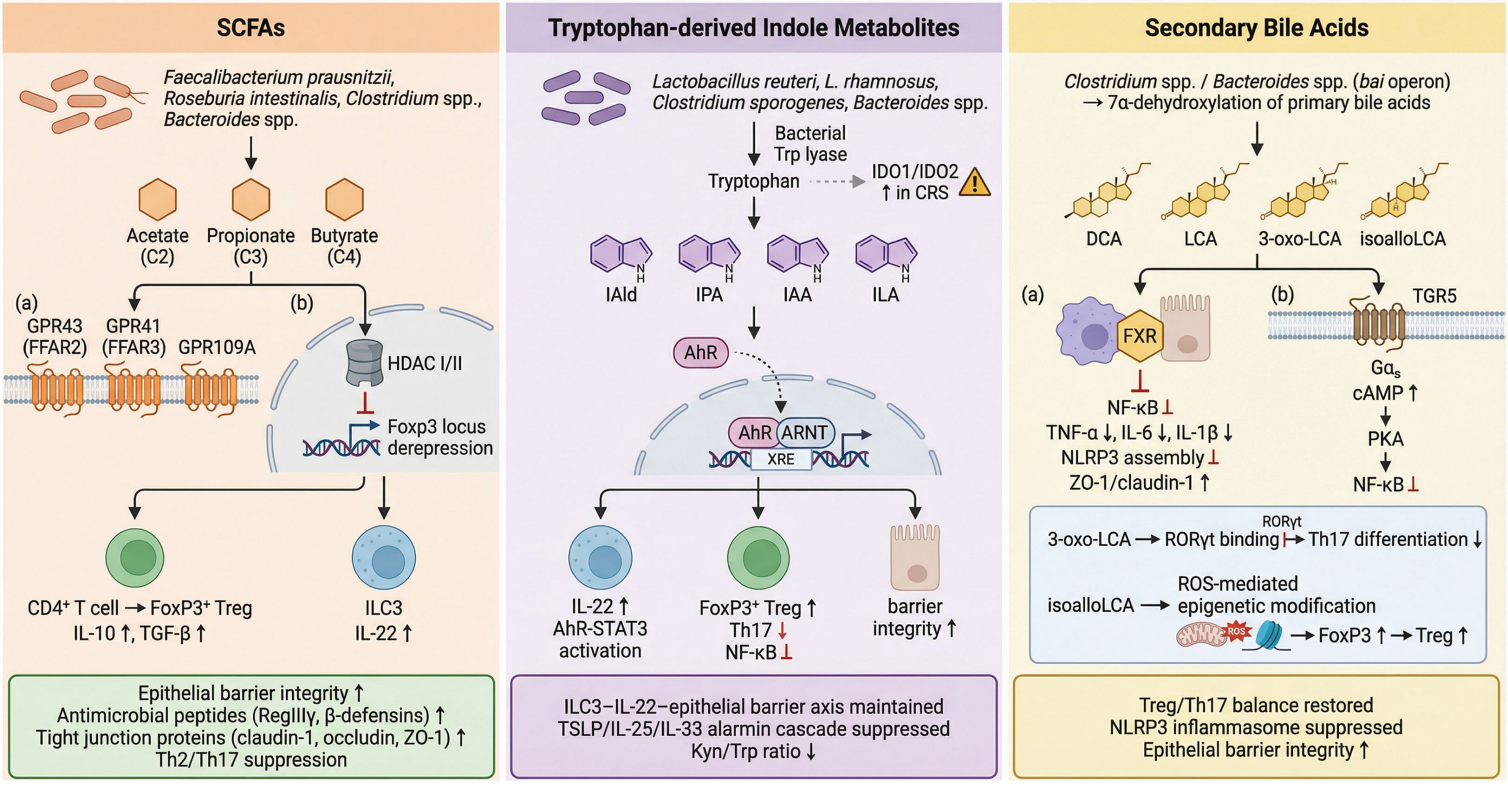
Molecular mechanisms of protective microbial metabolites in nasal mucosal immune regulation. This figure presents receptor-level signaling of three commensal-derived metabolite classes. SCFAs activate GPR43/GPR41/GPR109A and inhibit HDACs, inducing FoxP3+ Tregs, promoting ILC3-derived IL-22, and reinforcing epithelial tight junctions. Tryptophan-derived indoles engage AhR to sustain ILC3–IL-22 barrier protection. IDO1/IDO2 upregulation in CRS pathologically diverts tryptophan toward the kynurenine pathway. Secondary bile acids activate FXR (suppressing NLRP3/NF-κB) and TGR5 (cAMP-PKA). 3-oxo-LCA inhibits Th17 via RORγt. isoalloLCA induces FoxP3+ Tregs via mitochondrial ROS, collectively restoring Treg/Th17 balance. AhR, aryl hydrocarbon receptor. IDO, indoleamine 2,3-dioxygenase. LCA, lithocholic acid. FXR, farnesoid X receptor. TGR5, Takeda G protein-coupled receptor 5. NLRP3, NOD-like receptor family Pyrin domain containing 3. ROS, reactive oxygen species. HDAC, histone deacetylase. ILC3, group 3 innate lymphoid cell. Treg, regulatory T cell. RORγt, RAR-related orphan receptor gamma t.

Commensal bacteria in the healthy sinonasal and gut microbiome produce multiple classes of bioactive metabolites that function as immunological signals maintaining mucosal tolerance (17–19). Three classes have accumulated sufficient mechanistic and translational evidence to merit detailed discussion: SCFAs, tryptophan-derived indole metabolites, and secondary bile acids. Their key properties are summarized in **Table 1** and displayed in **Figure 2**.

**Table 1:** Summary of key protective microbial metabolites in nasal mucosal immune tolerance.

Metabolite class	Key molecules	Primary producing organisms	Key host receptors	Primary immune effects in nasal/mucosal immunity	CRS-relevant evidence	Evidence level	Strength of evidence
Short-Chain Fatty Acids (SCFAs)	Acetate (C2), Propionate (C3), Butyrate (C4)	*F. prausnitzii*, *Roseburia intestinalis*, *Clostridium* spp., *Bacteroides* spp.	GPR43 (FFAR2), GPR41 (FFAR3), GPR109A, HDACs	FoxP3+ Treg induction via HDAC inhibition; ILC3-IL-22 axis activation; NF-κB suppression; epithelial tight junction reinforcement; NLRP3 inflammasome inhibition; IgA class switching promotion	Reduced SCFA-producing commensals in CRS dysbiosis; butyrate supplementation decreases allergen-specific IgE and type 2 cytokines in allergic rhinitis models; deficiency correlates with CRS severity	Mechanistic (*in vitro*/animal) and human observational studies	Strong
Tryptophan-Derived Indole Metabolites	IAld (indole-3-aldehyde), IPA (indole-3-propionic acid), IAA (indole-3-acetic acid), ILA (indole-3-lactic acid)	*Lactobacillus reuteri*, *L. rhamnosus*, *Clostridium sporogenes*, *Bacteroides* spp.	AhR (aryl hydrocarbon receptor), PXR	AhR-ILC3-IL-22 barrier protection; FoxP3+ Treg induction; Th17 suppression via RORγt; antimicrobial peptide induction (RegIIIγ); NF-κB and NLRP3 inhibition by IAld; IDO1-mediated kynurenine pathway counter-regulation	Depletion of indole-producing commensals shifts Trp toward kynurenine pathway; elevated IDO activity/Kyn/Trp ratio in CRSwNP; AhR deficiency impairs ILC3-IL-22 signaling; IAld decreases inflammation in colitis models	Mechanistic (*in vitro*/animal) and human observational studies	Moderate
Secondary Bile Acids (SBAs)	DCA (deoxycholic acid), LCA (lithocholic acid), 3-oxo-LCA, isoalloLCA, UDCA	*Clostridium* spp., *Bacteroides* spp. (via 7α-dehydroxylation; *bai* operon)	FXR (farnesoid X receptor), TGR5 (GPBAR1), AhR	Treg/Th17 balance (3-oxo-LCA inhibits Th17 via RORγt; isoalloLCA promotes FoxP3+ Tregs via mitochondrial ROS); NLRP3 suppressionvia FXR; NF-κB inhibition via TGR5-cAMP-PKA axis; IgA production promotion; tight junction enhancement	Secondary bile acid deficiency promotes mucosal inflammation; SBA metabolism correlates with Treg numbers in mucosal sites; SBA derivatives regulate Treg responses relevant to eosinophilic CRSwNP endotype	Mechanistic (*in vitro*/animal) and human observational studies	Emerging
*S. aureus* Virulence Factors (Pathogenic)	Superantigens (SEA-SEE, TSST-1), Alpha-hemolysin (Hla), PSMα, Sphingomyelinase	*Staphylococcus aureus*	MHC-II/TCR Vβ (superantigens), NLRP3 inflammasome, toll-like receptors	Polyclonal T cell activation; type 2 immune polarization; local IgE class switching; macrophage pyroptosis via NLRP3-caspase-1-GSDMD; IL-1β and IL-18 release; epithelial tight junction (ZO-1, claudin-1, occludin) degradation	Dominant pathobiont in CRSwNP; superantigen-specific IgE in nasal polyp tissue; alpha-toxin disrupts nasal epithelial barrier; S. aureus enrichment inversely correlates with commensal protective metabolite levels	Mechanistic (*in vitro*/animal) and human observational studies	Emerging

IAld, indole-3-aldehyde; IPA, indole-3-propionic acid; IAA, indole-3-acetic acid; ILA, indole-3-lactic acid; DCA, deoxycholic acid; LCA, lithocholic acid; UDCA, ursodeoxycholic acid; AhR, aryl hydrocarbon receptor; FXR, farnesoid X receptor; TGR5, Takeda G protein-coupled receptor 5; GPR43, G protein-coupled receptor 43; HDAC, histone deacetylase; ILC3, group 3 innate lymphoid cell; Treg, regulatory T cell; NLRP3, NOD-like receptor protein 3; GSDMD, gasdermin D; PSM, phenol-soluble modulin; ROS, reactive oxygen species; PKA, protein kinase A; cAMP, cyclic adenosine monophosphate.

### 3.1 Short-chain fatty acids: GPR43 signaling, HDAC inhibition, and Treg induction

SCFAs, principally acetate (C2), propionate (C3), and butyrate (C4), are the primary fermentation products of dietary fiber and resistant starch by anaerobic bacteria including *Faecalibacterium prausnitzii*, *Roseburia intestinalis*, *Clostridium* species, and *Bacteroides* species, in both the gut and locally in the sinonasal microenvironment (12, 38). In healthy individuals, these metabolites are produced at concentrations sufficient to engage three primary host receptor systems: GPR43 (FFAR2), GPR41 (FFAR3), and GPR109A on immune and epithelial cells (18, 39–41). In addition, butyrate and propionate inhibit class I and II HDACs, providing an epigenetic dimension to SCFA-mediated immune regulation.

The immunological consequences of SCFA signaling are most profound at the level of CD4+ T cell differentiation. The landmark studies by Furusawa et al. and Arpaia et al., published simultaneously in Nature in 2013, established that commensal-derived butyrate is necessary and sufficient to drive FoxP3 expression in colonic CD4+ T cells, generating FoxP3+ Tregs (42, 43). The underlying mechanism involves HDAC inhibition at the Foxp3 locus and operates independently of TCR-mediated activation, distinguishing it from conventional antigen-driven T cell differentiation pathways. Smith et al. further demonstrated that all three major SCFAs regulate colonic Treg homeostasis through GPR43 (44). Subsequent mechanistic work by Park et al. showed that SCFAs simultaneously induce both regulatory and effector T cell subsets depending on dose and context, promoting immune balance through suppression of HDAC activity and regulation of mTOR-S6K signaling (45). Importantly, this Treg-inducing program is operative across multiple mucosal sites, and in the context of nasal mucosal immunity, SCFAs drive the differentiation of peripheral Tregs that, upon recruitment to sinonasal tissue, actively suppress Th2 and Th17 effector responses through IL-10 and TGF-β production (39, 46–48).

Beyond T cells, SCFAs directly regulate ILC3 responses (a cell population of critical importance to nasal mucosal barrier homeostasis). Yang et al. demonstrated that gut microbiota-derived SCFAs drive IL-22 production from ILC3s through GPR43 signaling (46), while Chun et al. identified that FFAR2 (GPR43) specifically regulates colonic ILC3 numbers and functional output (49). IL-22 produced by ILC3s activates STAT3 in nasal epithelial cells (50). This signaling cascade induces the expression of antimicrobial peptides (RegIIIγ, beta-defensins) and tight junction proteins (claudin-1, occludin, ZO-1) and promotes mucus production, collectively reinforcing the physical and chemical barriers against microbial penetration. Sepahi et al. further demonstrated that dietary fiber metabolites specifically regulate ILC responses through receptor-dependent mechanisms, emphasizing the direct link between dietary SCFA precursors and mucosal ILC homeostasis (51). Mann et al. recently synthesized the multi-dimensional regulatory network through which SCFAs link diet, the microbiome, and mucosal immunity, emphasizing the relevance of this axis to upper respiratory allergic and inflammatory conditions (52).

Clinical evidence supporting SCFA deficiency in CRS is accumulating. Kim and Baker comprehensively reviewed that SCFAs reduce eosinophil activity, suppress ILC2-driven airway hyperresponsiveness, and promote Treg development in murine models of allergic rhinitis and asthma, establishing SCFAs as active negative regulators of type 2 mucosal inflammation (17). Roduit et al. demonstrated in a prospective human birth cohort that high fecal butyrate and propionate levels in infancy are associated with significantly reduced atopy risk in childhood, providing clinical validation of the SCFA-mucosal tolerance axis (53). The depletion of butyrate-producing commensals that consistently accompanies CRS-associated dysbiosis thus plausibly contributes to impaired Treg induction and unconstrained type 2 immune activation in the nasal mucosa (15).

It is important to distinguish between SCFA mechanisms directly validated in airway contexts and those extrapolated from gut models. The foundational Treg-induction and ILC3-IL-22 evidence was established in colonic models (42). Direct nasal and airway validation, however, is increasingly available for certain aspects of SCFA biology. Immunohistochemical studies have confirmed the expression of GPR41 and GPR43 in human sinonasal tissue including nasal polyp epithelium (54). Functional studies further demonstrated that SCFAs induce tissue plasminogen activator expression in primary human nasal epithelial cells through these receptors, directly establishing the relevance of SCFA receptor signaling in the CRS nasal epithelial context. At the airway epithelial level, Richards et al. showed that butyrate and propionate restore barrier function in human bronchial epithelial cells, increasing transepithelial resistance and ZO-1 expression while reducing IL-4-induced IL-6 secretion (55). Wang et al. further demonstrated that sodium butyrate suppressed nasal TSLP expression and improved allergic rhinitis symptoms *in vivo* (56). By contrast, the regulation of ILC3 numbers and IL-22 output in sinonasal tissue, and the relative contribution of local versus systemic SCFA delivery to nasal Treg induction, remain inferred from gut models and constitute priority areas for direct nasal validation.

Beyond their immunomodulatory functions, commensal-derived SCFAs directly suppress *S. aureus* growth and virulence through complementary antibacterial mechanisms. Propionate and butyrate disrupt *S. aureus* branched-chain fatty acid metabolism, resulting in impaired growth, diminished agr quorum sensing expression, and increased sensitivity to membrane-targeting antimicrobials (57). Given that the agr system governs coordinated production of superantigens, alpha-hemolysin, and PSMs central to CRS pathogenesis, SCFA-mediated agr suppression constitutes a direct virulence attenuation mechanism within the sinonasal niche. *In vivo*, propionate ameliorated skin infections caused by MRSA by reducing cytokine production, bacterial loads, and abscess size (58). At the biofilm level, sodium butyrate significantly downregulated the mRNA expression of *S. aureus* invasion and biofilm formation-related genes ClfB and SdrC (59). These antibacterial effects are SCFA-species-specific and concentration-dependent, with propionate demonstrating the most consistent anti-staphylococcal potency, reinforcing the dual-mechanism rationale for propionate-enriched postbiotic formulations in CRSwNP.

### 3.2 Tryptophan metabolites: AhR–ILC3–IL-22 axis and barrier protection

Tryptophan (Trp) catabolism by gut and sinonasal commensal bacteria generates a structurally diverse family of indole-containing metabolites, including indole-3-aldehyde (IAld), indole-3-acetic acid (IAA), indole-3-propionic acid (IPA), and indole-3-lactic acid (ILA) (60, 61). These metabolites are generated predominantly through the indole pathway, catalyzed by bacterial Trp lyase and aromatic amino acid aminotransferases. Upon generation, they serve as potent endogenous agonists of the aryl hydrocarbon receptor (AhR), a promiscuous ligand-activated transcription factor that integrates environmental, dietary, and microbiota-derived cues to coordinate mucosal immune homeostasis (62, 63).

AhR activation by indole derivatives in the nasal mucosal context orchestrates a coordinated anti-inflammatory immunological program. Zelante et al. established that microbial tryptophan catabolites engage AhR to produce IL-22 and balance mucosal reactivity in the context of fungal infection, demonstrating that the AhR–ILC3–IL-22 axis is a critical component of mucosal homeostasis (64). In the nasal mucosa, ILC3s, the principal cellular source of IL-22 in the airway, express functional AhR, and their IL-22 production capacity is critically dependent on sufficient indole metabolite supply from the local and systemic commensal microbiome (46, 65). IL-22 produced by AhR-activated ILC3s promotes epithelial barrier repair, induces production of antimicrobial peptides, and suppresses the TSLP-IL-25-IL-33 alarmin cascade that initiates type 2 immune responses in CRS (66, 67).

Scott et al. demonstrated through well-controlled gnotobiotic experiments that microbial tryptophan metabolites, particularly IAld, indole-3-pyruvate, and indole-3-ethanol, directly increase gut epithelial barrier integrity through AhR signaling, establishing the protective barrier function of this receptor circuit (68). Rothhammer et al. extended these findings across organ systems, demonstrating that microbial Trp metabolites modulate AhR signaling in astrocytes, microglia, and peripheral immune cells, illustrating the systemic immunoregulatory reach of this metabolite–receptor axis (62, 69). Bender et al. provided highly compelling evidence in *Cell* that intratumoral *Lactobacillus reuteri* releases IAld as a tryptophan catabolite that activates AhR on immune cells and reprograms their functional state, directly establishing a “metabolite-as-drug” framework applicable to mucosal immune regulation (70). The implication for nasal mucosal immunity is that commensal bacteria capable of tryptophan catabolism—including *Lactobacillus reuteri*, *Lactobacillus rhamnosus*, *Clostridium sporogenes*, and *Bacteroides* species—are essential producers of AhR ligands that sustain the ILC3–IL-22–epithelial barrier axis in the nasal cavity (65, 70, 71).

Critically, depletion of tryptophan-metabolizing commensals in CRS-associated dysbiosis shifts tryptophan catabolism away from the protective indole pathway toward the kynurenine pathway, mediated by host enzymes IDO1 (indoleamine 2,3-dioxygenase 1) and IDO2. The resulting elevation in kynurenine/tryptophan (Kyn/Trp) ratio reflects both local tryptophan depletion and generation of immunosuppressive kynurenine metabolites that impair effector T cell function (37, 61). Malczewski et al. reviewed that elevated IDO activity and Kyn/Trp ratio are robustly associated with impaired immune surveillance and poor inflammatory disease outcomes, mechanistic principles with direct relevance to the CRS mucosal context (37). Lamas et al. demonstrated that CARD9 deficiency, whose polymorphisms are associated with mucosal inflammatory diseases, impairs gut microbiota metabolism of tryptophan into AhR ligands, establishing the genetic basis for metabolite-dependent AhR pathway deficiency in intestinal inflammation (65). IPA warrants specific emphasis: among indole metabolites, IPA activates AhR-STAT3 signaling to drive IL-22 secretion, downregulates IL-6 through NF-κB inhibition, promotes FoxP3+ Treg proliferation, and inhibits Th17 differentiation (18, 62). These combined properties make IPA a leading postbiotic candidate for CRS.

The broader tryptophan receptor landscape extends beyond AhR. Chang PV highlighted that tryptophan metabolites activate additional receptors including orphan GPCR GPRC5A and the pregnane X receptor (PXR), with downstream effects on epithelial permeability and inflammatory gene regulation (19). This receptor promiscuity suggests that the immunological effects of indole supplementation may engage multiple parallel pathways for mucosal immune homeostasis.

As with SCFA biology, the foundational AhR-ILC3-IL-22 axis was established in gut-associated models (64), and the CARD9-tryptophan-AhR ligand axis was characterized in the context of intestinal inflammation (65). Direct airway validation has been demonstrated at the level of epithelial barrier function. Scott et al. established AhR-dependent barrier regulation by tryptophan metabolites using gnotobiotic intestinal models, while studies in lung and airway contexts have shown that indole-3-acetic acid promotes airway epithelial barrier repair through modulation of IL-22 expression, providing partial translational support (68). More recently, Wang et al. demonstrated in both *in vivo* COPD mouse models and *in vitro* airway macrophage models that indole-3-aldehyde activates AhR to inhibit the HDACs-NF-κB-NLRP3 signaling pathway (72). This intervention reduced TNF-α, IL-1β, and IL-6 levels and alleviated airway inflammation, directly validating the IAld-AhR-NLRP3 suppression axis in a respiratory disease context. Nevertheless, the specific capacity of IAld or IPA to restore ILC3-derived IL-22 production and normalize ILC subset balance in human sinonasal tissue has not yet been directly demonstrated in nasal-specific models and represents a mechanistic extrapolation from gut ILC3 biology.

Although both tryptophan-derived indole metabolites and secondary bile acids are capable of engaging the aryl hydrocarbon receptor, their receptor hierarchies and dominant downstream signaling programs are fundamentally distinct and must not be conflated. Most microbial tryptophan-derived metabolites are recognized as AhR-selective agonists, including indoxyl sulfate, indigo, indole-3-acetic acid, indole-3-aldehyde, and 3-methylindole, making AhR the organizing receptor node for their barrier-protective ILC3-IL-22 effects (73). In contrast, secondary bile acids DCA and LCA are preferential ligands for GPBAR1 (TGR5), while CDCA is the most potent FXR ligand, and TGR5 responds to bile acids in the order of TLCA, LCA, DCA, CDCA, and CA (74, 75). The dominant immunological outputs of LCA and DCA therefore flow through the TGR5-cAMP-PKA axis and FXR-mediated transcriptional suppression of NF-κB, with AhR representing only an accessory target. Nevertheless, the simultaneous availability of both metabolite classes in the nasal mucosal microenvironment may yield convergent and potentially synergistic AhR activation that amplifies Treg-inducing outputs beyond what either class achieves independently, representing a multi-metabolite mechanism of mucosal immune regulation warranting direct investigation.

### 3.3 Secondary bile acids: FXR, TGR5, and the Treg/Th17 balance

Secondary bile acids (SBAs), principally deoxycholic acid (DCA) and lithocholic acid (LCA), are generated in the colon through 7α-dehydroxylation of hepatic primary bile acids by anaerobic bacteria including Clostridium and Bacteroides species carrying the bai operon (76, 77). Although the nasal mucosa does not constitute a primary site of bile acid metabolism, SBAs enter the enterohepatic circulation and reach the systemic bloodstream, where they engage immune cells throughout the body (17, 78).

At physiological concentrations, SBAs activate two principal receptor systems: the nuclear receptor FXR (farnesoid X receptor) and the membrane-bound GPCR TGR5 (77, 78). FXR activation in mucosal macrophages, dendritic cells, and epithelial cells exerts pleiotropic anti-inflammatory effects (18, 77). Specifically, FXR suppresses NLRP3 inflammasome assembly, reduces transcription of proinflammatory cytokines (TNF-α, IL-6, IL-1β) through NF-κB inhibition, and enhances the expression of tight junction proteins (ZO-1, claudin-1) in the epithelium. TGR5 activation elevates intracellular cAMP through Gαs signaling, activating protein kinase A (PKA) to suppress NF-κB-mediated inflammatory gene expression in macrophages and DCs (78).

In the adaptive immune compartment, specific SBA derivatives exert highly targeted effects on CD4+ T cell differentiation directly relevant to the Treg/Th17 imbalance characteristic of severe CRS. Hang et al. demonstrated in *Nature* that 3-oxo-LCA inhibits Th17 differentiation by directly binding and suppressing RORγt, while isoalloLCA promotes FoxP3 induction in Tregs through reactive oxygen species-mediated epigenetic modifications (79). Campbell et al. showed that bacterial bile acid metabolites drive peripheral Treg generation in an FXR-dependent manner (80), and Song et al. further demonstrated that combinations of primary and secondary bile acids induce RORγt+ FoxP3+ Tregs in the colon (81). The SBA-Treg axis thus represents a powerful immunological mechanism through which gut microbial metabolism of cholesterol-derived compounds shapes adaptive immune tolerance at distal mucosal sites including the nasal cavity (17, 82). Furthermore, SBAs function as endogenous AhR ligands, suggesting a potential synergistic interaction with the tryptophan metabolite-AhR pathway, representing a convergent multi-metabolite mechanism of mucosal immune tolerance regulation (18, 83).

For secondary bile acids, the evidentiary basis remains most strongly rooted in gut and systemic immunological models, and the translation to the nasal mucosal context relies most heavily on the gut-nose metabolite axis framework. The landmark studies by Hang et al. demonstrating 3-oxo-LCA-mediated RORγt suppression and isoalloLCA-driven FoxP3 induction were conducted in colonic T cell populations (79). FXR and TGR5 receptor expression has been characterized in intestinal and hepatic tissues, and while systemic bile acid signaling can in principle engage these receptors in circulating immune cells that subsequently traffic to the nasal mucosa, direct receptor expression and functional signaling studies in sinonasal epithelial cells, sinonasal macrophages, or sinonasal T cell populations have not yet been reported. The mechanistic claims regarding secondary bile acid-mediated nasal mucosal Treg-Th17 balance regulation should therefore be understood as biologically plausible hypotheses grounded in systemic metabolite availability and receptor conservation, pending direct sinonasal validation.

The SBA-Treg/Th17 axis has particular therapeutic relevance to Asian CRS, where non-type 2 endotypes predominate (40–80% of CRSwNP patients) and the T3 endotype characterized by elevated IL-17A and IL-17F and neutrophilic inflammation is especially prevalent (84). In this context, 3-oxo-LCA inhibits Th17 differentiation through direct RORγt binding and inverse agonism, while isoalloLCA and isoDCA promote FoxP3+ Treg induction through mitochondrial ROS-mediated epigenetic modifications and enhanced Foxp3/IL-10/TGF-β expression (84) (85). For Asian CRS patients whose disease is driven primarily by Th17-neutrophilic axes rather than ILC2-eosinophilic axes, SBA derivatives, particularly 3-oxo-LCA and isoalloLCA, represent the most endotype-matched postbiotic candidates, offering the potential to simultaneously suppress pathogenic Th17 differentiation and augment Treg-mediated counter-regulation through distinct but complementary receptor mechanisms. This endotype-SBA alignment critically distinguishes the therapeutic framework for Asian non-type 2 CRS from the indole metabolite-AhR-ILC3 axis that is most relevant to Western-dominant type 2 CRSwNP.

## 4 Pathogenic microbial metabolites: mechanisms of immune tolerance disruption in CRS

**Figure 3:**
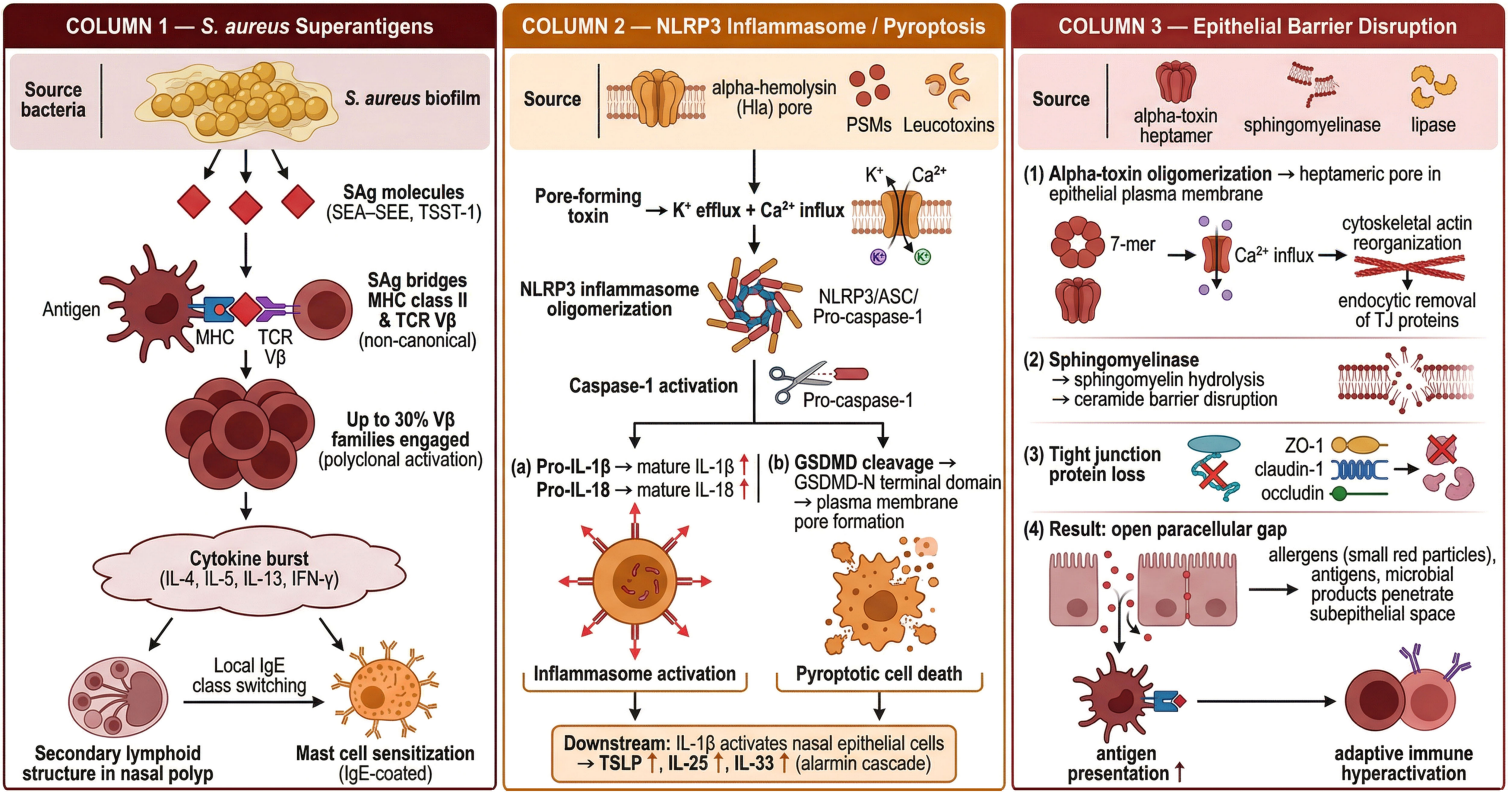
Molecular mechanisms of pathogenic microbial metabolites in nasal mucosal immune regulation. This figure delineates three parallel mechanisms by which *S. aureus* virulence factors disrupt nasal mucosal immune tolerance. Column 1 (Superantigens): SEA-SEE and TSST-1 crosslink MHC-II/TCR Vβ non-canonically, driving polyclonal T cell expansion, a type 2 cytokine burst (IL-4, IL-5, IL-13), and local IgE class switching within secondary lymphoid structures of nasal polyp tissue. Column 2 (NLRP3/Pyroptosis): Pore-forming toxins (Hla, PSMs) activate the NLRP3–ASC–caspase-1 axis, releasing IL-1β/IL-18, cleaving GSDMD to execute macrophage pyroptosis, and triggering epithelial alarmin production (TSLP, IL-25, IL-33). Column 3 (Barrier Disruption): Alpha-toxin heptamers and sphingomyelinase degrade tight junction proteins (ZO-1, claudin-1, occludin) and the ceramide lipid barrier, creating paracellular gaps that permit allergen/antigen penetration and adaptive immune hyperactivation. TSST-1, toxic shock syndrome toxin-1. NLRP3, NOD-like receptor family Pyrin domain containing 3. GSDMD, gasdermin D. Hla, alpha-hemolysin. PSM, phenol-soluble modulin. TSLP, thymic stromal lymphopoietin. ZO-1, zonula occludens-1.

While commensal-derived metabolites maintain nasal mucosal tolerance through the receptor signaling programs described above, metabolites and toxins produced by pathobiont organisms, most prominently Staphylococcus aureus, actively dismantle this homeostatic equilibrium through multiple parallel mechanisms and displayed in **Figure 3**.

### 4.1 *S. aureus* superantigens: non-canonical T cell activation and local IgE amplification

*S. aureus* produces a repertoire of superantigenic enterotoxins, including staphylococcal enterotoxins A through E and TSST-1 (86, 87). These toxins activate T cells through non-canonical engagement of MHC class II molecules and TCR Vβ regions, bypassing antigen-specific activation and driving polyclonal T cell expansion involving up to 30% of the total T cell pool. This superantigen-mediated polyclonal activation generates a substantial local cytokine burst (including IL-4, IL-5, IL-13, and IFN-γ) that promotes type 2 immune polarization in the sinonasal mucosa. Gevaert et al. demonstrated that nasal polyp tissue contains secondary lymphoid organ-like structures where local IgE class switching to *S. aureus* enterotoxins occurs, generating *S. aureus*-specific IgE that sensitizes local mast cells and amplifies inflammatory responses upon subsequent encounter (87). Kowalski et al. further confirmed that superantigen-specific IgE antibodies are associated with severe and refractory airway inflammatory disease, establishing the direct clinical relevance of *S. aureus* toxin–IgE biology to CRS pathogenesis (88).

### 4.2 NLRP3 inflammasome activation and macrophage pyroptosis

Beyond superantigens, S. aureus elaborates pore-forming toxins such as alpha-hemolysin (Hla), phenol-soluble modulins (PSMs), and bicomponent leucotoxins that activate the NLRP3 inflammasome in sinonasal macrophages and epithelial cells. NLRP3 activation drives caspase-1 cleavage, which processes pro-IL-1β and pro-IL-18 into their active forms and cleaves gasdermin D (GSDMD), generating plasma membrane pores that execute pyroptotic cell death (89, 90). The pyroptotic cascade has been mechanistically characterized at molecular resolution: Shi et al. (89) established GSDMD as the executioner protein of pyroptotic membrane lysis, while Kayagaki et al. (90) defined the non-canonical caspase-11/GSDMD axis.

In the CRS context, macrophage pyroptosis has a dual pathological impact. First, the IL-1β burst released by pyroptotic macrophages directly activates nasal epithelial cells to produce alarmin cytokines TSLP, IL-25, and IL-33 (10, 91). Second, repeated pyroptotic waves deplete the sinonasal macrophage pool available for tissue homeostasis and bacterial clearance, perpetuating the dysbiotic state. As comprehensively reviewed by Zeng et al., the interplay between pathogen-derived metabolites and toxins with the NLRP3 inflammasome represents a central node in escalating mucosal inflammatory cycles (18). The opposing regulatory actions of protective SCFA-mediated NLRP3 suppression and pathogenic *S. aureus* toxin-mediated NLRP3 activation thus constitute a critical metabolite-regulated immunological checkpoint in CRS.

### 4.3 Epithelial barrier disruption by *S. aureus*-derived toxins

*S. aureus* virulence factors directly disrupt the epithelial barrier through multiple biochemical mechanisms. Alpha-toxin oligomerizes to form heptameric pores in the plasma membrane of nasal epithelial cells, inducing calcium influx, cytoskeletal reorganization, and endocytic removal of tight junction proteins, particularly claudin-1, occludin, and ZO-1 (88, 92). The resultant loss of paracellular seal integrity allows luminal antigens, allergens, and microbial products to penetrate into the subepithelial space, substantially expanding antigen presentation and adaptive immune activation (11, 13). *S. aureus* additionally produces sphingomyelinase and lipase enzymes that hydrolyze the sphingomyelin component of the epithelial membrane, further compromising the ceramide-based lipid barrier that maintains paracellular impermeability (88).

Therefore, the evidence reviewed in this section indicates that CRS pathogenesis involves a bidirectional metabolite imbalance. Commensal-derived protective metabolites (SCFAs, indole derivatives, SBAs) are simultaneously depleted while pathobiont-derived toxins and immune-disruptive metabolites are enriched. As Kim and Baker comprehensively summarized, this dual dysregulation compromises barrier cell function and regulatory immune cell activity while amplifying proinflammatory signaling, creating a self-reinforcing cycle of epithelial barrier collapse, antigen penetration, alarmin release, and type 2 immune escalation (17).

### 4.4 Fungal metabolites and bacterial-fungal cross-kingdom metabolic interactions in CRS immune tolerance disruption

Allergic fungal rhinosinusitis (AFRS), a subset of CRSwNP characterized by antifungal IgE sensitivity, eosinophil-rich mucus, and characteristic imaging findings, develops in immunocompetent patients and is driven by adaptive and innate type 2 immune responses (93). A deficiency of histatins impairs the clearance of fungal spores, leading to fungi-induced disruption of the epithelial barrier and stimulation of sinonasal epithelial cells. These events trigger a cascade of type 2 inflammatory cytokines mediated by both the adaptive and innate immune systems. Tyler et al. highlighted that this involves loss of antimicrobial peptides, increased epithelial permeability, and fungal protease-driven release of innate inflammatory cytokines including IL-25, IL-33, and TSLP, with fungi-activated receptors including protease-activated receptor 2 (PAR2) and TLR4 driving release of these epithelial-derived alarmins (30).

At the molecular level, fungal serine proteases represent the primary biochemical agents through which fungi disrupt nasal mucosal barrier integrity. Alternaria enhances the production of reactive oxygen species (ROS) and reduces transepithelial resistance (TER), while decreasing the mRNA and protein expression of tight junction molecules including ZO-1, occludin, and claudin-1 in nasal epithelial cells (94). Critically, pretreatment of Alternaria with a serine protease inhibitor or heat inactivation restored ROS levels, TER, and tight junction expression to those of unstimulated controls, confirming the protease-dependence of these barrier-disrupting effects. This protease-dependent barrier disruption converges mechanistically with the alarmin cascade described for S. aureus toxins in Section 4.3, establishing that both bacterial and fungal pathobionts exploit tight junction degradation as a shared immunological gateway to amplify type 2 inflammation.

Beyond protease-mediated barrier disruption, Aspergillus fumigatus elaborates a portfolio of bioactive secondary metabolites that actively suppress mucosal innate immune defenses. Gliotoxin (GT), a secondary metabolite and major virulence factor of Aspergillus fumigatus, broadly suppresses host innate immune responses (95). Among the most extensively studied fungal secondary metabolites, aflatoxins, ochratoxin, and gliotoxin are the best characterized for immunosuppressive activity, and notably, in all cases these compounds mainly affect innate macrophage and neutrophil responses, especially the pro-inflammatory response, highlighting the importance of these cells in the elimination and prevention of Aspergillus infection (96). GT is a well-characterized immunosuppressive mycotoxin that targets the first-line epithelial immune defense of the host and has been shown to induce apoptosis in leukocytes and to inhibit phagocytosis, respiratory burst, and T and B cell responses (97). The mechanistic analogy to S. aureus virulence factors is instructive: whereas S. aureus alpha-hemolysin activates NLRP3 inflammasome-mediated pyroptosis to deplete the sinonasal macrophage pool (Section 4.2), Aspergillus-derived gliotoxin independently depletes this same innate sentinel population through apoptotic and necrotic pathways, creating a convergent fungal-bacterial mechanism of innate immune suppression in the sinonasal niche (98).

Candida albicans contributes a mechanistically distinct class of immunomodulatory fungal metabolites through the quorum-sensing molecule farnesol, a sesquiterpene alcohol generated by enzymatic dephosphorylation of farnesyl diphosphate during fungal biofilm growth. Farnesol mediates cross-species and even cross-kingdom communication, playing a pivotal role in the interactions of C. albicans with its host, the microbiome, and competing pathogens (99). Critically, farnesol exerts potent immunomodulatory effects on host dendritic cells (DCs), the professional antigen-presenting cells responsible for orchestrating adaptive immune responses to fungal and bacterial antigens in the sinonasal mucosa. Farnesol affects the sentinels of the immune system: using primary human monocytes differentiating into DCs, farnesol was found to alter the expression of antigen presentation and maturation markers, reduce the secretion of IL-12 while increasing the expression of IL-10, and impair the ability of DCs to prime T cells (99). This farnesol-induced DC reprogramming represents a metabolite-mediated mechanism of adaptive immune tolerance subversion that is highly relevant to the CRS context: the reduction of DC-derived IL-12 impairs Th1 antifungal responses while the increase in IL-10 creates a tolerogenic DC phenotype that may paradoxically permit persistent fungal colonization while simultaneously suppressing Treg-mediated counter-regulation of type 2 immunity.

The cross-kingdom dimension of CRS pathogenesis is most sharply illustrated by the interaction between C. albicans-derived farnesol and S. aureus, the dominant bacterial pathobiont in CRSwNP. Farnesol is a quorum sensing molecule secreted by C. albicans and shown to play a central physiological role during fungal biofilm growth. Kong et al. characterized an intricate interaction between C. albicans and S. aureus in co-existing biofilm communities (100). The molecule secreted by C. albicans inhibits the synthesis of staphyloxanthin, a pigment considered an important virulence factor in S. aureus, while transcriptional analysis demonstrated that this secreted molecule also modulates the expression of virulence-associated genes in S. aureus (101). When fungi and *S. aureus* are co-inoculated in the sinonasal mucosa, robust polymicrobial biofilm formation is observed, and the resulting mucosal inflammation and tissue injury are more severe than those produced by bacterial or fungal inoculation alone (29). These cross-kingdom biofilm interactions represent a metabolite-mediated mechanism through which the simultaneous enrichment of both fungal and bacterial pathobionts in CRS dysbiosis amplifies mucosal immune disruption beyond what either organism could achieve independently. The fungal-bacterial interaction in the sinonasal mucosa affects microbial survival, virulence, and host immune responses. The interplay between the mycobiome and bacteriome further shapes local inflammation and disease chronicity through these shared immunological pathways.

## 5 The gut–nose metabolite axis: systemic determinants of nasal mucosal immunity

### 5.1 Gut-derived metabolites as systemic immune regulators

The sinonasal mucosal immune system does not function in immunological isolation. Rather, it is continuously influenced by gut-derived metabolites that reach the systemic circulation through the portal vein and thoracic duct (18, 102, 103). Gut-derived SCFAs directly shape the phenotype of circulating monocytes, NK cells, and T cells, promoting anti-inflammatory and regulatory states that are subsequently maintained when these cells traffic to the nasal mucosa (39, 52). Trompette et al. demonstrated that gut microbiota metabolism of dietary fiber produces SCFAs that modulate hematopoiesis in the bone marrow (104). These SCFAs skew myeloid precursor differentiation toward anti-inflammatory dendritic cells and macrophages with reduced capacity to drive allergic airway responses. This SCFA-hematopoiesis-mucosal immunity circuit provides a mechanistic explanation for epidemiological observations linking Western diet, characterized by low fiber intake and reduced SCFA production, with increased CRS and allergic rhinitis risk (17, 104).

Tryptophan metabolites follow an analogous systemic route. IPA produced by gut *Lactobacillus* species and *Clostridium sporogenes* enters the portal circulation and is detectable in peripheral blood, where it engages AhR in circulating immune cells (36, 62). Converging evidence from multiple studies demonstrates that gut-derived microbial metabolites shape respiratory mucosal immunity through the gut-lung axis, and that antibiotic-induced gut dysbiosis amplifies airway inflammatory responses through depletion of protective metabolite signals (105, 106). Kim and Baker further reviewed evidence that SCFAs reaching the nasal mucosa through systemic circulation suppress allergic rhinitis in murine models by inhibiting ILC2 activity and promoting Treg expansion, directly validating the clinical relevance of the gut–nose metabolite axis (17).

### 5.2 Bidirectionality and clinical implications

Emerging evidence suggests the gut-nose relationship is bidirectional: nasal inflammatory conditions may alter gut microbiome composition through swallowing of postnasal secretions containing inflammatory mediators, systemic cytokine signaling, and shared mucosal immune circuits (102, 105). CRS patients consistently demonstrate alterations in gut microbiome composition, including depletion of SCFA-producing *Faecalibacterium prausnitzii* and enrichment of potentially pathogenic Proteobacteria (15, 104). Tan et al. demonstrated that dietary fiber and SCFA supplementation promote oral and systemic immune tolerance through multiple cellular pathways, supporting the therapeutic logic of gut microbiome modulation for distant mucosal inflammatory conditions (107). As reviewed by Zeng et al., the dysbiosis–immune axis operates across multiple organ systems through shared metabolite-receptor signaling pathways, implying that restoring gut microbiome metabolite production could yield downstream benefits for sinonasal immune regulation (18).

### 5.3 Dietary modulation of the gut–nose metabolite axis

Diet represents the most proximate modifiable determinant of gut microbial metabolite production. High dietary fiber intake promotes proliferation of SCFA-producing bacteria including *Faecalibacterium prausnitzii*, *Roseburia intestinalis*, and *Akkermansia muciniphila*, resulting in elevated systemic SCFA concentrations that confer nasal mucosal protection (39, 52). Conversely, Western dietary patterns suppress SCFA-producing commensals, increase secondary bile acid accumulation toward proinflammatory species, and favor immune activation over tolerance (17). Wastyk et al. demonstrated that gut microbiota-targeted diets modulate human immune status in a metabolite-dependent manner, establishing the immunological efficacy of dietary microbiome interventions (108). These findings collectively position dietary modification as both a preventive and adjunctive therapeutic strategy for nasal mucosal immune regulation.

### 5.4 Geographic heterogeneity of gut microbiome metabolite production and its implications for CRS endotype distribution

The geographic distribution of CRS inflammatory endotypes reflects the profound influence of diet and environment on gut microbiome metabolite production capacity. While Western CRS is predominantly type 2, Asian populations show remarkable heterogeneity: over 50% of Chinese CRSwNP cases are non-eosinophilic and more than 73% of Korean CRS cases are non-type 2 or mixed endotypes (109). Non-type 2 Th17-dominant inflammation is the state most sensitive to secondary bile acid-mediated Treg/Th17 rebalancing, whereas type 2 ILC2-dominant inflammation is most amenable to SCFA-driven NLRP3 suppression and indole metabolite-driven AhR-ILC3 restoration.

The increasing prevalence of lifestyle-related diseases in Asia has been associated with dietary changes that alter gut microbiota composition and metabolic function (109). The penetration of modern Western dietary patterns into Asia, characterized by increased fat content and decreased plant-derived dietary fiber, is progressively restructuring the Asian gut microbiome. Traditional Asian dietary patterns, characterized by high intake of plant-based fermented foods, dietary fiber, and fermented soybean products, favor the proliferation of anaerobic bacteria carrying the bai operon necessary for secondary bile acid 7α-dehydroxylation. This microbial enrichment maintains a bile acid pool composition enriched in the protective derivatives 3-oxo-LCA, isoalloLCA, and isoDCA, thereby supporting Treg/Th17 homeostasis. Conversely, comparative metabolomics in Asian children showed that those consuming more traditional plant-based diets had higher levels of short-chain fatty acids, while those consuming more urbanized diets had lower SCFA levels and higher amino acid levels. This dietary-metabolite relationship mechanistically connects the adoption of Western dietary patterns to simultaneous depletion of both SCFA and protective SBA signals, providing a metabolite-level explanation for the emerging pattern of immune dysregulation observed in Asian urban populations.

## 6 Postbiotics as a novel therapeutic strategy: restoring nasal mucosal immune homeostasis

### 6.1 Definition and therapeutic rationale

**Figure 4:**
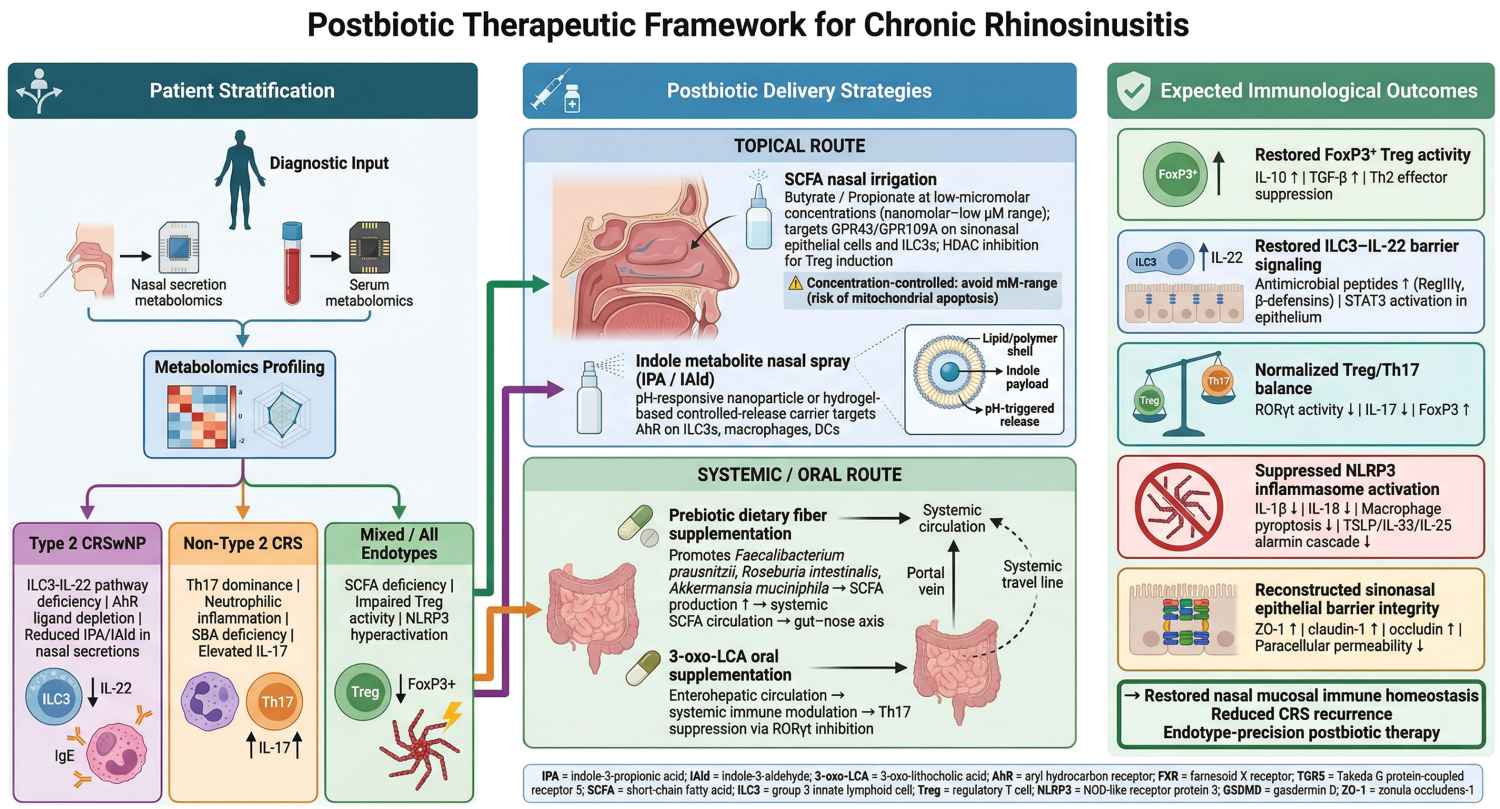
Postbiotic therapeutic strategies targeting the microbial metabolite–immune axis in CRS. This figure presents an endotype-guided postbiotic therapeutic framework for CRS. Left (Patient Stratification): Nasal and serum metabolomics stratify three endotypes: type 2 CRSwNP, non-type 2 CRS, and mixed/all endotypes. Central (Delivery): Topical routes include SCFA nasal irrigation and indole metabolite spray via pH-responsive nanoparticles; systemic/oral routes include prebiotic fiber supplementation (gut-nose SCFA axis) and oral 3-oxo-LCA (RORγt inhibition). Right (Outcomes): Therapy is projected to restore FoxP3+ Treg activity, recover ILC3-IL-22 barrier signaling, normalize Treg/Th17 balance, suppress NLRP3 activation, and reconstruct epithelial barrier integrity, restoring nasal mucosal homeostasis and reducing CRS recurrence. SCFA, short-chain fatty acid. 3-oxo-LCA, 3-oxolithocholic acid. ILC3, group 3 innate lymphoid cell. Treg, regulatory T cell. NLRP3, NOD-like receptor family Pyrin domain containing 3. RORγt, RAR-related orphan receptor gamma t.

The International Scientific Association of Probiotics and Prebiotics (ISAPP) formally defined postbiotics as “preparations of inanimate microorganisms and/or their components that confer a health benefit on the host” (110, 111). Within this framework, purified microbial metabolites, including specific SCFAs, indole derivatives, and bile acid derivatives, represent the most mechanistically well-characterized subclass (**Figure 4**). Compared with probiotic supplementation using live bacteria, metabolite-based postbiotics offer critical advantages for clinical translation: precise and reproducible dosing, absence of colonization or infection risk, pharmaceutical-grade stability, and the ability to target specific receptor–signaling pathways at defined concentrations (110, 111). Maslowski et al. established GPR43 as a key SCFA receptor driving anti-inflammatory immune regulation, directly establishing the molecular basis for SCFA-based postbiotic therapy (112). The therapeutic rationale for postbiotic intervention in CRS is mechanistically direct: if disease perpetuation involves simultaneous depletion of protective metabolite-receptor signals and enrichment of pathogenic metabolite signals, direct supplementation of the former should restore homeostatic receptor activation.

### 6.2 SCFA postbiotics: topical and systemic delivery strategies

The anti-inflammatory utility of butyrate and propionate supplementation has been demonstrated in multiple mucosal inflammatory diseases. Roduit et al. showed that high fecal butyrate and propionate levels in early life are associated with protection against atopy (69), and Lewis et al. demonstrated that dietary fiber-induced SCFA production suppresses ILC2-dependent airway hyperresponsiveness, directly relevant to the CRS type 2 endotype (113). Kim and Baker reviewed that administration of butyrate in conjugated starch formulations decreases serum allergen-specific IgE, reduces IL-4 and IL-5 production, and improves clinical activity in murine models of allergic rhinitis (17).

For CRS applications, topical SCFA delivery via nasal irrigation represents a mechanistically compelling but technically demanding route, as engaging GPR43 and GPR109A directly on sinonasal epithelial cells and resident ILC3s could promote local Treg differentiation, NLRP3 inflammasome suppression, and ILC3-derived IL-22 production within the sinonasal mucosa (52, 112). As Zeng et al. comprehensively reviewed, the dose-dependent effects of SCFAs must be precisely controlled, given that nanomolar to low micromolar butyrate concentrations promote epithelial repair and Treg induction through GPR109A and HDAC inhibition, while millimolar concentrations can activate mitochondrial apoptosis and compromise barrier integrity (18, 114). Salvi and Cowles similarly emphasized the concentration threshold dependency of butyrate effects on intestinal epithelium, underscoring the need for controlled-release delivery formulations in postbiotic design (114). pH-responsive nanoparticle or hydrogel-based systems achieving controlled SCFA release within the nasal cavity represent a promising pharmaceutical engineering strategy to navigate this therapeutic window (21, 111).

Three pathological barriers impose fundamental constraints on the effective delivery of topically administered postbiotic metabolites in CRS. The first barrier is mucociliary clearance (MCC), a critical physiological defense mechanism of the respiratory tract. MCC drives the rapid clearance of nasally administered substances from the nasal cavity to the nasopharynx, thereby substantially reducing drug absorption following intranasal application. Under normal conditions, MCC operates at approximately 8 mm per minute, with normal mucociliary transit ranging between 12 and 15 minutes (115). For conventional aqueous formulations, this results in a clearance half-life of approximately 15 minutes, and mucoadhesion allowing prolonged retention time is therefore often considered a prerequisite for effective nasal administration (116). In CRS, chronic type 2 inflammation leads to goblet cell hyperplasia and loss of ciliated epithelial differentiation, generating a more tenacious and diffusion-resistant mucus layer that entraps conventionally delivered molecules before meaningful epithelial contact is established. The second barrier is fibrotic stromal remodeling of the polyp tissue. CRSwNP is associated with inflammation and tissue remodeling including myofibroblast differentiation and extracellular matrix deposition mediated by TGF-β1 and IL-4, with the most described ECM components being collagen 1A1 and 1A2 alongside infiltration of inflammatory cells (117). Furthermore, tissue remodeling caused by increased MMPs is involved in the pathogenesis of CRSwNP, and elevated periostin and tenascin C within the polyp stroma constitute a dense physical diffusion barrier that postbiotic metabolites must traverse to reach subepithelial immune cell populations including ILC3s, Treg precursors, and tissue-resident macrophages (118). This diffusion resistance is expected to disproportionately impede hydrophilic free SCFAs relative to more lipophilic indole derivatives. The third barrier is microcirculatory dysfunction. VEGF, expressed at markedly elevated levels in nasal polyp tissue (sevenfold higher in nasal lavage than in control subjects, induces endothelial proliferation and vascular hyperpermeability, generating elevated interstitial fluid pressure that opposes convective inward flux of topically applied molecules and limits subepithelial metabolite penetration (119, 120). Thus, these three barriers constitute a coherent argument that topical postbiotic delivery in CRS demands substantially more sophisticated formulation strategies than conventional corticosteroid sprays, as postbiotic metabolites must achieve receptor-activating concentrations at subepithelial immune populations within a structurally remodeled tissue environment marked by microcirculatory dysfunction. These considerations reinforce the translational rationale for mucus-penetrating PEG-coated nanoparticle systems, mucoadhesive thermosensitive hydrogels, and postoperative delivery strategies exploiting the improved anatomical access achieved following endoscopic sinus surgery.

### 6.3 Indole metabolite postbiotics: AhR agonism for ILC3 restoration

Among tryptophan-derived postbiotic candidates, IPA and IAld have the most favorable mechanistic profiles for nasal mucosal applications. As reviewed by Zeng et al., IAld suppresses NF-κB activation and NLRP3 inflammasome assembly, promotes FoxP3+ Treg proliferation, and inhibits Th17 differentiation through AhR signaling (18). In the nasal mucosal context, AhR agonism by IPA or IAld would be expected to restore ILC3-derived IL-22 production, repair the epithelial barrier, normalize tight junction protein expression, and rebalance the ILC1/ILC3 ratio toward the homeostatic ILC3-dominant state (64, 65). The capacity of IAld to simultaneously activate AhR in multiple immune cell populations, including ILC3s, macrophages, and DCs, positions it as a pleiotropic immunoregulatory postbiotic for CRS (70, 83).

Quintana et al. established that AhR is a critical molecular switch controlling the Treg/Th17 balance: AhR activation promotes FoxP3+ Treg generation and simultaneously limits RORγt-driven Th17 differentiation, providing the molecular basis for AhR-agonist postbiotics as precision Treg-inducing agents (63). The “metabolite-as-drug” framework established by Bender et al. and mechanistically elaborated by Chang PV provides a rigorous scientific basis for pharmaceutical-grade development of indole metabolite postbiotics, bypassing the need for viable bacteria while achieving receptor-targeted immune modulation (19, 70).

### 6.4 Endotype-guided precision postbiotic therapy

A principal insight derived from the metabolite-immune axis framework is that postbiotic selection should be guided by patient endotype and individual metabolomics profiling. Type 2 CRSwNP, characterized by ILC2 hyperactivation, eosinophilic infiltration, and impaired ILC3-IL-22 signaling, represents the primary target for indole metabolite-based AhR agonist postbiotics that restore ILC3 activity and normalize the ILC1/ILC2/ILC3 balance (10, 65). Non-type 2 CRS, characterized by Th1/Th17 dominance and neutrophilic inflammation, may respond preferentially to bile acid derivatives, specifically 3-oxo-LCA, that directly suppress Th17 differentiation through RORγt inhibition (79). SCFA-based postbiotics confer broader benefits across CRS endotypes through their capacity to simultaneously promote Treg induction, ILC3 activation, and NLRP3 suppression (39, 45).

Metabolomics profiling of nasal secretions and serum could identify specific metabolite deficiencies in individual patients, enabling personalized postbiotic prescription. Malczewski et al. reviewed that patient-specific metabolite signatures predict treatment responses and guide immunological interventions in cancer immunotherapy (37). This principle is directly translatable to CRS: nasal secretion metabolomics could identify SCFA deficiency, reduced AhR ligand availability, or SBA dysregulation as the primary driver of immune tolerance breakdown in individual patients.

### 6.5 Physicochemical and formulation considerations for intranasal postbiotic delivery

SCFAs are small, highly polar molecules that are rapidly dispersed and cleared by nasal fluid before meaningful epithelial contact is established (121). This rapid clearance necessitates mucoadhesive excipients such as chitosan or poloxamer-based thermosensitive *in situ* gels, which transition from sol to gel at physiological temperature and substantially prolong mucosal residence time. Indole metabolites including IPA and IAld, despite moderate lipophilicity, are susceptible to oxidative and enzymatic degradation in nasal secretions, and encapsulation within liposomes or nanoemulsions protects these compounds from inactivation while enhancing epithelial penetration through lipid bilayer-mediated transcellular transport (122). Secondary bile acid derivatives, being more hydrophobic, are amenable to nanostructured lipid carrier or liposomal formulation strategies. For all three metabolite classes, the established concentration-dependent cytotoxic thresholds mandate controlled-release engineering to maintain receptor-activating concentrations, and postoperative endoscopic access provides a clinically actionable window for initial optimized mucoadhesive postbiotic deposition.

## 7 Conclusions and future perspectives

CRS research is undergoing a fundamental conceptual transition from documenting microbial compositional dysbiosis to deciphering the metabolite–receptor mechanisms through which the microbiome communicates with the nasal mucosal immune system. The mechanistic evidence synthesized in this review establishes a coherent microbial metabolite-immune receptor axis. This axis encompasses four interconnected signaling modules: SCFA-GPR43/HDAC-Treg induction, indole metabolite-AhR-ILC3-IL-22 barrier protection, secondary bile acid-FXR/TGR5-Treg/Th17 balance regulation, and *S. aureus* toxin-NLRP3 inflammasome-pyroptosis-barrier disruption. This axis is bidirectionally regulated by both local sinonasal and systemic gut microbiome-derived metabolites, creating a distributed immunological circuit that either maintains or fails to maintain nasal mucosal tolerance.

Several critical scientific questions require resolution to advance this framework from concept to clinical practice. First, the quantitative metabolite landscape of the healthy sinonasal microenvironment, specifically the physiological concentrations of SCFAs, indole metabolites, and SBAs in nasal secretions, requires systematic characterization using validated metabolomics workflows, as these data will inform the therapeutic target concentrations for postbiotic formulations. Second, comprehensive single-cell transcriptomic mapping of metabolite receptor expression (GPR43, AhR, FXR, TGR5) in sinonasal epithelial cells, ILC subsets, and resident T cells across CRS endotypes is needed to identify cell-type-specific metabolite–receptor interactions that determine disease outcome. Third, controlled longitudinal studies with matched gut and nasal microbiome and metabolomics data are essential to resolve the causal directionality of the gut-nose axis in CRS. Fourth, clinical pharmacology studies of topically delivered SCFA and indole metabolite formulations in the nasal cavity must characterize absorption, local concentration profiles, and dose-response relationships, particularly given the concentration-dependent dual effects of these metabolites on epithelial cell survival and immune activation.

Prospectively, integration of AI-driven metabolite-receptor interaction prediction with patient-level multi-omics data holds promise for identifying novel postbiotic candidates within the vast chemical space of microbial metabolites that remains uncharacterized. A fundamental limitation of conventional nasal secretion metabolomics is the chemical superimposition of host- and microbial-derived metabolites without spatial attribution. We propose integrating spatial metabolomics with single-cell sequencing to resolve this ambiguity directly within the polyp microenvironment. Frameworks such as metaFISH and scSpaMet enable co-localization of metabolite signals with cell-type-specific transcriptional signatures, establishing mechanistically grounded, spatially explicit models of postbiotic signaling in CRS. The convergence of precision postbiotic design, endotype-specific patient stratification, and mechanism-guided delivery technology ultimately has the potential to reframe CRS management from chronic symptom suppression with systemic immunosuppressants toward targeted restoration of the ecological and immunological balance that defines nasal mucosal health.
